# Conceptions of Happiness and Unhappiness among Italian Psychology Undergraduates

**DOI:** 10.1371/journal.pone.0167745

**Published:** 2016-12-15

**Authors:** Igor Sotgiu

**Affiliations:** Department of Human and Social Sciences, University of Bergamo, Bergamo, Italy; University of New South Wales, AUSTRALIA

## Abstract

The present study aims at investigating the conceptions of happiness and unhappiness in a sample of Italian psychology undergraduates. Participants completed a questionnaire asking them to report the most important things that made them feel happy (*happiness components*) and those ones that made them feel unhappy (*unhappiness components*). Different measures of overall happiness and overall unhappiness were also obtained by asking respondents to assess to what extent each reported happiness and unhappiness component was present in their life, and by inviting them to provide a global judgment about their happiness and unhappiness. Results indicated that participants did not conceptualize happiness and unhappiness as perfect antonyms. Indeed, both investigated concepts encompassed a similar set of semantic components; however, the perceived salience of some of them − assessed in terms of frequency of citation and average ranking − significantly varied between happiness and unhappiness. With regard to the measurement of overall happiness and unhappiness, on average, respondents considered themselves as moderately happy and only slightly unhappy. However, a judgmental asymmetry was found when comparing global and specific evaluations of unhappiness. Theoretical and empirical implications of the study are discussed.

## Introduction

“*There is nothing more vague than the idea of happiness*, *that old prostituted*, *adulterated word so full of poison that we would like to exclude it from the language*”. [1]

Many, if not all, happiness researchers would agree with the first part of Pascal Bruckner’s quotation, stating that happiness is a concept with fuzzy boundaries, whose meaning varies somewhat across individuals, languages, cultures, as well as historical periods (see, for example, [2–11]). However, almost surely, the majority of them would not agree with the last part of the French writer’s quotation. Indeed, eliminating the word *happiness* from the human language would be a mistake for at least two reasons. First, since concepts are mental representations of objects, living beings, and events [12–13], removing the word *happiness* would not imply the elimination of the concept associated to it. This means that people could continue to form and use symbolic representations of happiness even in absence of a specific term indicating this concept. Second, there is empirical evidence indicating that *happiness* is a word used very frequently in everyday language. In a study conducted on a sample of 200 Canadian English-speaking undergraduates, Fehr and Russell [14] asked participants to mention items belonging to the category “emotion” and to stop after about one minute or 20 items. Results showed that happiness (in its various syntactic variants) was the most frequently cited emotional category, being reported by 76% of participants. Subsequent studies conducted in samples of non English-speaking participants [15–18] confirmed that *happiness* is one of the most used emotion words, even if its frequency of usage varies widely across languages.

The present article deals with the meaning components of the happiness and unhappiness concepts within the Italian cultural and linguistic context. While most happiness scholars devoted and are currently devoting their efforts to develop empirical indicators of how citizens from various countries feel happy and satisfied with their lives (for reviews, see [19–23]), in recent years several researchers have started to investigate the *folk* psychology of happiness, namely how common people represent the concept of happiness and its semantic space. Empirical studies conducted in this field targeted participants from different countries and continents. In the following, I summarize the main findings from this literature, paying special attention to research investigating the Italian cultural context.

The first study on the folk psychology of happiness was conducted by Lu [24]. This researcher asked 142 Chinese undergraduates to write free-format essays in response to this simple question: “What is happiness?”. Lu then coded the participants’ essays using the thematic analysis. Results yielded to the identification of four main themes, which can be summarized as follows: 1) Happiness is a complex mental process encompassing positive emotions and an optimistic approach towards the future; 2) happiness is a harmonious state of existence where the individual maintains a positive relationship with oneself and with the external world; 3) happiness and unhappiness are locked in a relationship of mutual influence; 4) there are some virtues (e.g., gratitude, generosity, transcendence) which are essential to achieve happiness. Overall, these results were interpreted by the author as reflecting the collectivistic orientation of the Chinese culture.

In a series of subsequent questionnaire studies, Galati, Sotgiu, and colleagues [25–27] investigated samples of citizens from the Italian and the Cuban population, including adults, older adults, as well as young students. Subjects who took part in these investigations were administered open-ended questions asking them to list the things that made them feel happy. In addition, researchers asked participants to order these things according to their perceived importance. The categorization of participants’ responses collected across studies and age groups allowed the authors to identify a varying number of semantic components of happiness (comprised between 20 and 25), the great majority of which were found in both investigated cultural groups. With reference to the Italian samples, *health*, *family*, and *money* were the most cited components. Since these components were generally assessed by the Italian participants as the most important ones, Galati and colleagues considered them as the core attributes defining the happiness concept within the Italian cultural context (cf. [25–26]).

In another study by Delle Fave, Brdar, Freire, Vella-Brodrick and Wissing [28], 666 adult participants (age 30–51 years) from seven countries (Australia, Croatia, Germany, Italy, Portugal, Spain, and South Africa) were asked to answer the open-ended question “What is happiness for you?”. Participantsʼ definitions of happiness were coded by the researchers and then quantitatively analyzed with reference to the entire sample. Results showed that the definitions referred to both life domains and psychological dimensions, which were equally distributed in percentage. More in detail, the most reported life domains were, in order, *family*, *interpersonal relationships*, and *health*. On the other hand, the psychological dimensions most frequently cited were *harmony*, *positive emotions*, and *well-being*, respectively.

More recently, Delle Fave et al. [29] implemented the same research methodology in a larger study involving 2799 adults from 12 countries. Importantly, this study also separately analyzed and reported data collected from a sample of 216 Italian citizens. Overall, the happiness definitions provided by these participants were semantically consistent with the happiness definitions obtained from the international sample previously examined by Delle Fave et al. [28], and also with the happiness components found in the Italian studies by Galati, Sotgiu, and colleagues [25–27]. Indeed, the life domains reported most frequently by these participants were, in order, *interpersonal relationships*, *family*, and *health*. Moreover, the most reported psychological dimension was *harmony*, followed by *satisfaction*, *optimism*, and *positive emotions*.

Taken together, the studies reviewed above show that the happiness concept covers a wide range of semantic components, including life domains, emotional experiences, socially shared beliefs, as well as the fulfillment of material and psychological basic needs. It is important to note that, although these studies significantly contributed to identify the prototypical attributes of the happiness concept in a number of countries, much more could have been learnt if they also would have investigated the semantic structure of the antonym concept: i.e., unhappiness. Indeed, since the foundation of the Stoic school, many philosophers and social scientists have assumed that happiness and unhappiness represent intertwined constructs (see [22, 30]), being connected at both the semantic level and the experiential level. For instance, with regard to the latter point, it is conceivable that people pursuing a happy life would avoid those things that make them unhappy. At the same time, people keeping away from an unhappy life are expected to strive for and attain those things that make them happy.

Based on what has been said so far, psychological research jointly investigating the folk concepts of happiness and unhappiness is strongly recommended. However, to the best of my knowledge, there is only one study in the psychological literature following this approach. This is the quali-quantitative investigation by Uchida and Kitayama [31], who examined the lay conceptions of happiness and unhappiness among American and Japanese undergraduates. In the first part of this study, participants from both cultural groups were asked to describe up to five characteristics of either happiness or unhappiness. In a second step, features generated by participants were printed on separate index cards, which constituted the experimental material to be used with new groups of undergraduates from both countries. Specifically, these participants were given a stack of cards from their own culture with the instruction to sort the cards into meaningful groups, namely taking into consideration the similarities between the descriptions printed on the cards. Sorting data were then analyzed by means of multidimensional scaling, which allowed Uchida and Kitayama to obtain separate “mental maps” for happiness and unhappiness in form of two-dimensional diagrams. Besides the differences between the two investigated cultural groups, results showed that the participants’ representations of happiness encompassed five broad semantic categories: *Positive hedonic experiences*, *personal achievement*, *social harmony*, *transcendental reappraisal*, and *social disruption*. On the other hand, the semantic categories defining the unhappiness concept were *negative hedonic experiences*, *cognitive appraisal* (including the two subcategories *personal failure* and *social disruption*), *transcendental reappraisal*, *self-improvement*, and *externalizing behavior*.

Overall, Uchida and Kitayamaʼs [31] findings suggest that the concepts of happiness and unhappiness were not represented by participants as perfect antonyms. Indeed, while the mental maps obtained for these concepts referred to similar themes (i.e., hedonic experiences, personal achievement, social relationships), the unhappiness concept encompassed two distinctive semantic components, which mainly referred to personal strategies to cope with suffering (i.e., *self-improvement* and *externalizing behavior*). Importantly, the generalizability of these conclusions should be tested in other cultural contexts, as well as using different methods to analyze the subjective conceptions of both happiness and unhappiness. The present work moves in that direction.

## The Present Study: Aims and Hypotheses

This quali-quantitative study extends previous work by Galati, Sotgiu, and colleagues [25–27], by jointly investigating the conceptions of both happiness and unhappiness in a sample of Italian undergraduates. In particular, the primary goal of this investigation was to compare the participantsʼ representations of happiness and unhappiness, identifying the common and distinctive components of meaning defining these two concepts and assessing their perceived importance. A second aim of the study was to build empirical indicators of overall happiness and overall unhappiness directly reflecting the participants’ representations of the components defining these two concepts. The significance of these indicators was also assessed by comparing them with more traditional self-report measures of happiness and unhappiness.

Based on the study by Uchida and Kitayama [31] described in the introduction, it was predicted that participantsʼ representations of happiness and unhappiness would encompass both similarities and differences. However, given the exploratory nature of the present investigation, no hypotheses were formulated about the specific semantic components defining these similarities and differences.

## Method

### Design and Procedure

This study followed a within-subjects design, as all participants were asked to fill in a questionnaire which investigated their lay concepts of both happiness and unhappiness. All participants were recruited from two introductory courses in Psychology at the University of Bergamo (Northern Italy). Importantly, both courses took place during the first semester of the participants’ first study year and data were collected at the very beginning of these courses.

The data reported in the current article were analyzed anonymously and the review board of the University of Bergamo approved this study. Questionnaires were administered in classrooms under the supervision of the author of this article. Participants were told that the research was concerned with their beliefs about happiness and unhappiness. They were also informed that their answers would be used for research purposes only. The respondents were not paid for their participation. One hundred eighty questionnaires were distributed: Of these, two were excluded as they were returned either largely incomplete or without sociodemographic information. The response rate was thus 98.9%.

### Participants

One hundred and seventy-eight undergraduate psychology students (29 males, 149 females) took part in the study. The age of participants ranged from 18 to 36 years, with a mean age of 20.5 years (*SD* = 3.44). All respondents were Italians and lived in the Lombardy region of Northern Italy at the time of the investigation.

### Questionnaire

The questionnaire had two sections, which were administered in a counterbalanced order across respondents. All but one item constituting the first section were taken from the questionnaire used in the Galati et al.’s study [25]. More specifically, in this section, participants were asked to think about their idea of happiness and to write down at least five things that made them feel happy (*happiness components*). To measure the level of attainment of each of the reported happiness components, respondents were then asked to evaluate, on an 11-point rating scale, to what extent they had attained them in their lives (0 = *not at all*, 10 = *totally*). Finally, a new item asked participants to estimate, on an 11-point rating scale, their level of global happiness (“*Please*, *think about your life as a whole*. *How much do you feel happy*?”, 0 = *not at all*, 10 = *totally*). The second section of the questionnaire was designed specifically for this study and aimed at exploring the participants’ lay concepts of unhappiness using the same set of questions employed in the first section. In particular, respondents were asked to think about their idea of unhappiness and to write down at least five things that made them feel unhappy (*unhappiness components*). Participants were then asked to evaluate, on an 11-point rating scale, to what extent each reported unhappiness component was currently present in their lives (0 = *not at all*, 10 = *totally*). As in the first section, the participants’ level of global unhappiness was also assessed using an 11-point rating scale (“*Please*, *think about your life as a whole*. *How much do you feel unhappy*?”, 0 = *not at all*, 10 = *totally*). Importantly, in both sections of the questionnaire, respondents were explicitly asked to report the components they thought about in order of relevance, namely starting with the component they considered as most important and ending with the one deemed as least important.

## Results

### The Categorization of Happiness and Unhappiness Components

On average, the participants reported 5.59 (*SD* = 1.57, range 3–15) happiness components and 5.50 (*SD* = 1.31, range 3–12) unhappiness components. According to paired samples *t* test, these values were not statistically different, *t*(177) = 0.84, *p* = .40. Moreover, *t* test for independent samples showed that the number of both happiness and unhappiness components did not significantly vary as a function of the gender of participants (happiness: *t*(30) = 0.77, *p* = .44; unhappiness: *t*(31) = 0.61, *p* = .55).

The happiness and unhappiness components mentioned by the participants were content analyzed and grouped into categories according to a criterion of semantic similarity. The categorization was jointly performed by the author of the present article and a licensed psychologist, who together determined the criteria to be followed when coding the participants’ responses and resolved disagreements through discussion. We first coded the happiness components reported by the study participants and then the unhappiness components. As for the former, the starting point of the categorization work was the study by Galati et al. [25], in which 21 categories of happiness components were identified. With the exception of only one of the previously found categories (i.e., *home*), all the other 20 categories were used also in the present study.

This set of categories was extended with the following six happiness components: *Goals*, *environmental mastery*, *autonomy*, *self-knowledge*, *pets*, and *luck*. Importantly, the first three of these new categories were included as they allowed us to establish a connection between participants’ beliefs about their happiness and recent theoretical advances in the understanding of this construct within Positive Psychology (e.g., [32–36]). However, the category *self-knowledge* well reflected the typical inclinations and attitudes of psychology students. Examples of the responses included in this category were “knowing myself”, “being aware of my personal weaknesses”, and “being able to reflect on my negative experiences”. Finally, *pets* and *luck* completed the list of 26 happiness components found in the present study (see Table 1). Noteworthy, the labels, as well as the contents, of four categories of happiness components identified in the Galati et al.’s [25] study were slightly modified in the present investigation. Based on empirical studies and theoretical work by Peterson and Seligman [32], the category *values* was renamed as *values and virtues*. Furthermore, the categories *good affective relationships*, *hobbies*, and *culture and knowledge* were renamed as *good social relationships*, *hobbies and interests*, and *knowledge/education*, respectively.

**Table 1:** Frequency (in Percentage), Mean Rank, Salience, and Mean Attainment Scores of Happiness Components

Happiness Component	Frequency *%*	Rank *M* (*SD*)	Salience index	Attainment*M* (*SD*)
Family	59.0	2.20 (1.37)	0.4687	7.59 (2.34)
Friendship	55.1	2.54 (1.16)	0.4010	7.88 (1.39)
Love	47.8	2.36 (1.37)	0.3643	6.81 (2.84)
Health	46.1	2.99 (1.60)	0.2934	8.38 (1.25)
Self-actualization	33.7	3.17 (1.44)	0.2012	6.67 (1.91)
Good social relationships	29.2	2.77 (1.52)	0.1987	7.58 (1.65)
Success	33.7	3.58 (1.53)	0.1885	6.30 (2.19)
Self-knowledge	20.2	3.47 (2.02)	0.1186	6.94 (2.25)
Money	30.3	4.65 (1.33)	0.1171	5.85 (2.06)
Values and virtues	16.9	3.80 (2.02)	0.0968	7.00 (2.49)
Goals	18.5	3.64 (1.80)	0.0957	6.30 (2.37)
Partner	12.4	2.36 (1.26)	0.0932	7.64 (2.94)
Hobbies and interests	20.8	4.32 (1.40)	0.0901	7.24 (1.98)
Serenity/well-being	12.4	3.27 (2.12)	0.0802	6.14 (1.93)
Positive emotions	10.7	3.32 (1.49)	0.0662	7.84 (1.30)
Pleasant events	13.5	4.08 (1.74)	0.0659	7.04 (2.16)
Work	10.7	4.11 (1.29)	0.0515	5.21 (3.08)
Knowledge/education	11.2	4.30 (1.56)	0.0489	7.35 (1.60)
Helping others	9.0	4.19 (1.38)	0.0401	8.06 (1.73)
Environmental mastery	6.2	3.82 (1.40)	0.0312	6.55 (1.21)
Autonomy	6.2	4.36 (1.96)	0.0283	6.91 (1.30)
Absence of unpleasant events	3.4	4.83 (1.33)	0.0117	5.83 (2.23)
Faith	2.2	4.50 (3.00)	0.0116	8.75 (0.96)
Pets	2.2	5.75 (2.87)	0.0076	9.00 (1.15)
Luck	2.2	5.25 (2.06)	0.0071	4.25 (2.75)
Sex	1.1	4.50 (0.71)	0.0041	8.50 (0.71)

*Note*. Happiness components are ordered by salience index, from high to low.

As for the unhappiness components, the categorization work took advantage of the results of the coding of happiness components. A total of 25 categories of unhappiness components were identified (see Table 2). Of these, 21 categories were semantically equivalent to those found in the coding of happiness responses, namely they referred to the same core theme. They were: *Health problems*, *bad social relationships*, *failure*, *family problems*, *lack of self-actualization*, *negative emotions*, *love problems*, *negative values and personal weaknesses*, *money problems*, *lack of self-knowledge*, *unpleasant events*, *work-related problems*, *difficulties in achieving goals*, *friendship difficulties*, *lack of environmental mastery*, *depression* (which can be considered the opposite of *serenity/well-being*), *lack of knowledge/education*, *lack of autonomy*, *problems with the partner*, *lack of hobbies and interests*, and *sexual dissatisfaction*. Importantly, when comparing the entire list of categories of unhappiness components with that one of happiness components, it is also possible to identify some components which referred to only one of the two investigated concepts. The distinctive components of unhappiness found in the present study were *death*, *loneliness*, *interpersonal loss*, and *insecurity*. On the other hand, the distinctive components of happiness were *helping others*, *absence of unpleasant events*, *faith*, *pets*, and *luck*. It should be recognized that the classification of *loneliness* as a distinctive component of the unhappiness concept is questionable. Indeed, the meaning of this component partially overlaps with the one of other two semantic categories which were mentioned by participants, but cannot be considered as distinctive unhappiness components (i.e., *bad social relationships*, *friendship difficulties*). With this respect, I note that the decision to consider *loneliness* as a semantically distinct unhappiness component was based on the qualitative analysis of participants’ free responses, which revealed that respondents referred to the abstract concept of loneliness without giving any further specifications.

**Table 2:** Frequency (in Percentage), Mean Rank, Salience, and Mean Presence in Life Scores of Unhappiness Components

Unhappiness Component	Frequency *%*	Rank *M* (*SD*)	Salience index	Presence in life *M* (*SD*)
Death	39.3	1.53 (0.86)	0.3533	4.12 (3.71)
Loneliness	43.8	2.71 (1.68)	0.3109	3.42 (3.06)
Health problems	44.4	2.73 (1.46)	0.3015	2.84 (2.84)
Bad social relationships	39.3	3.33 (1.53)	0.2296	4.25 (2.87)
Failure	37.6	3.57 (1.33)	0.2008	3.63 (2.78)
Family problems	27.5	2.78 (1.48)	0.1867	4.62 (2.84)
Lack of self-actualization	24.2	2.98 (1.57)	0.1542	4.17 (2.83)
Negative emotions	25.3	3.40 (1.56)	0.1489	5.44 (2.72)
Love problems	23.0	3.54 (1.82)	0.1352	4.65 (3.08)
Negative values and personal weaknesses	22.5	3.38 (1.71)	0.1338	4.90 (3.16)
Money problems	30.3	4.15 (1.19)	0.1318	4.11 (2.53)
Interpersonal loss	17.4	2.55 (1.41)	0.1238	4.33 (3.76)
Lack of self-knowledge	20.8	3.43 (1.67)	0.1186	4.70 (3.13)
Unpleasant events	15.2	3.30 (1.88)	0.0949	5.00 (2.92)
Work-related problems	15.2	3.30 (1.20)	0.0901	4.04 (3.85)
Difficulties in achieving goals	15.2	3.15 (1.49)	0.0885	3.85 (2.71)
Friendship difficulties	14.0	3.12 (1.36)	0.0848	3.52 (2.76)
Lack of environmental mastery	12.4	3.45 (1.41)	0.0688	3.41 (2.44)
Insecurity	10.1	3.83 (1.62)	0.0512	5.22 (2.90)
Depression	6.2	3.27 (2.05)	0.0383	2.73 (2.19)
Lack of knowledge/education	7.3	4.23 (1.01)	0.0369	3.54 (3.20)
Lack of autonomy	5.6	3.60 (1.27)	0.0318	3.80 (3.05)
Problems with the partner	2.8	3.40 (1.14)	0.0157	6.00 (3.08)
Lack of hobbies and interests	1.7	5.67 (2.08)	0.0057	3.67 (1.53)
Sexual dissatisfaction	1.1	5.50 (0.71)	0.0021	4.00 (5.66)

*Note*. Unhappiness components are ordered by salience index, from high to low.

In order to verify the reliability of both the happiness and unhappiness components’ categories, three final-year psychology students–who were unaware of the aims of the study–were asked to codify the responses provided by the participants using the 26 categories of happiness components and the 25 categories of unhappiness components identified in the first stage of the analysis. The correspondence between the categorization performed by the researchers and that done by each of the naive judges was then calculated using Cohen’s *k*. All *k* values were greater than .84 (*p* < .05 in all cases), thus indicating good inter-rater agreement.

### Frequency, Mean Rank, and Salience of Happiness and Unhappiness Components

For each component of happiness and unhappiness, I computed the percentage of participants who cited it and the mean rank of its order of mention. Moreover, the overall perceived importance of each component was assessed by means of the salience index proposed by Smith and Borgatti [37]. This statistical index, also called *Smith’s salience index*, takes into consideration the following three parameters: i) the number of respondents citing a specific semantic component; ii) the component’s average ranking; iii) the length of the lists of components in which the target component was cited by the participants. Specifically, I used the following formula (corrected by Sutrop [38], p. 269):

$$S=(\sum ((L_{i}-R_{i}+1)/L_{i}))\;/N$$

where *S* is the salience of the component, *L*_*i*_ is the length of the lists of components in which the target component *j* was cited, *R*_*j*_ is the rank given by respondents to the target component, and *N* is the total number of participants. Noteworthy, the Smith’s salience index ranges from 0 to 1, with higher scores indicating greater salience of the component.

Frequency of mentioning, mean ranks, and salience indexes computed for all the happiness and unhappiness components are presented in Tables 1 and 2, respectively. As can be seen, the most salient happiness components were, in order, *family*, *friendship*, *love*, *health*, and *self-actualization*. On the other hand, the most salient unhappiness components were *death*, *loneliness*, *health problems*, *bad social relationships*, and *failure*, respectively. The salience scores for the components of happiness and unhappiness referring to the same themes were compared using paired samples *t* tests. Results showed that participants considered family, *t*(177) = 7.99, *p* < .001, friendship, *t*(177) = 10.61, *p* < .001, love, *t*(177) = 6.20, *p* < .001, hobbies and interests, *t*(177) = 5.74, *p* < .001, and partner, *t*(177) = 3.91, *p* < .001, as themes more salient for their happiness than for their unhappiness. By contrast, everyday emotions, *t*(177) = 3.28, *p* = .001, and environmental mastery, *t*(177) = 2.40, *p* = .02, were judged by participants as themes more salient for their unhappiness than for their happiness.

### Semantic Maps of Happiness and Unhappiness

In order to further analyze the relationship between the frequency with which happiness and unhappiness components were reported and their perceived importance, correspondence analyses were computed separately for two investigated concepts. Following the same statistical strategy used by Sotgiu et al. [27], these analyses were performed on two contingency tables in which the rows were the happiness and unhappiness components, respectively, and the columns were the orders given to the components (from 1 to 5) by the participants. The scores in the cells of these cross-tabulations thus indicate the number of participants who cited a component in a specific order within a specific concept. Importantly, only those components which were reported in the top five positions by at least 10 participants were retained for the analyses. This data reduction determined the exclusion of six happiness components (*autonomy*, *absence of unpleasant events*, *faith*, *pets*, *luck*, *sex*) and four unhappiness components (*lack of autonomy*, *problems with the partner*, *lack of hobbies and interests*, *sexual dissatisfaction*). As a result, a 20×5 and a 21×5 cross-tabulations constituted the two final matrixes on which correspondence analyses were computed.

**Fig 1:**
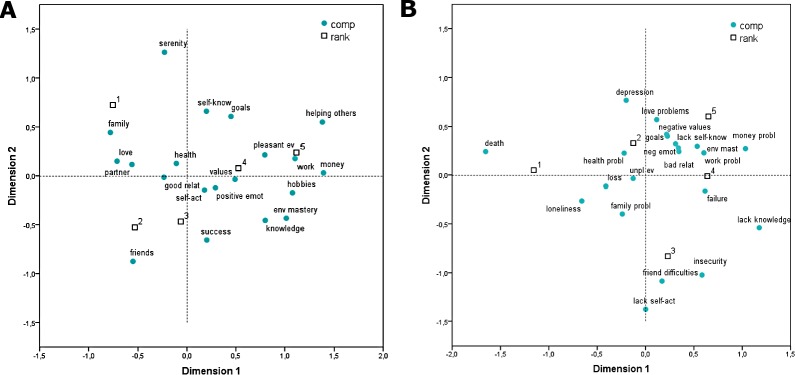
Semantic maps of happiness and unhappiness. (A) Correspondence analysis map of frequency and order of importance of happiness components. (B) Correspondence analysis map of frequency and order of importance of unhappiness components. *Note*. The numbers from 1 to 5 indicate the rank of components given by participants.

Fig 1A and 1B show, respectively, the correspondence analysis maps of happiness and unhappiness, which were obtained by crossing the first and the second dimension extracted (the symmetrical method was chosen as normalization procedure). The total amount of inertia explained by these dimensions was 78.8% for happiness and 82.9% for unhappiness. As can be seen in Fig 1A, three main groups of happiness components can be identified. A first group, which occupies the top left hand part of the graph, includes *family*, *love*, and *partner*. *Family* and *love*, in particular, appear closer to the first rank symbol, indicating that they were frequently cited in this position by the participants. Between the lower left and right hand quadrants, there is a second group of components including *friendship* and *success*, which are closer to the second and third ranks, respectively. A third cluster of components can be identified between the upper and lower right quadrants, in proximity of the fourth and fifth ranks. Components belonging to this cluster significantly vary as far as their semantic content is concerned. More in detail, they include *values and virtues*, *pleasant events*, *work*, *money*, *hobbies and interests*, *environmental mastery*, and *knowledge/education*. The remaining happiness components are positioned closer to the graph origin or in other places of the map which are distant from the rank symbols.

Three groups of components can also be identified when looking at the unhappiness map displayed in Fig 1B. A first group, appearing in the upper and lower left hand quadrants, include *death* and *loneliness*. Both components are positioned at a similar distance from the first rank, thus confirming that they were frequently cited in this position by participants. A second group of components appears in the lower right hand quadrant. This group includes *friendship difficulties*, which is closer to the third rank, *insecurity* and *lack of self-actualization*. Finally, a third group of components can be identified between the upper and the lower right quadrants, where the fourth and fifth ranks appear. The semantic content of components belonging to this cluster is quite heterogeneous. They include *failure*, *work-related problems*, *bad social relationships*, *negative emotions*, *lack of self-knowledge*, *lack of environmental mastery*, and *money problems*. As in the happiness map, there are some unhappiness components placed closer to the graph origin or in other areas of the map which are distant from the rank symbols. Lastly, the second rank was closer to the graph origin.

### Measuring Happiness and Unhappiness Taking into Account their Subjective Conceptions

The last column of Tables 1 and 2 presents the mean attainment/presence in life scores for each component defining happiness and unhappiness, respectively. Considering only the 10 most salient components identified within the two investigated concepts, the happiness components attained most were *health* (*M* = 8.38, *SD* = 1.25) and *friendship* (*M* = 7.88, *SD* = 1.39), with mean values around 8. On the other hand, the unhappiness components most present in participants’ lives were *negative emotions* (*M* = 5.44, *SD* = 2.72) and *negative values and personal weaknesses* (*M* = 4.90, *SD* = 3.16); however, the mean scores associated with the latter components were much lower, namely around the midpoint of the rating scale.

The analysis of mean global ratings of happiness (*M* = 6.98, *SD* = 1.59) and unhappiness (*M* = 3.37, *SD* = 2.02) revealed that participants considered themselves as moderately happy and only slightly unhappy. A further index of overall happiness and one of overall unhappiness were computed separately by calculating, for each participant, the mean of the attainment/presence in life scores attributed by respondents to their happiness and unhappiness components, respectively. The values of these two indicators–called *composite index of overall happiness* and *composite index of overall unhappiness*–were averaged across all respondents and then compared with mean global ratings of happiness and unhappiness.

Mean scores computed for the various measures of overall happiness and overall unhappiness are reported in Table 3, which also displays the results of pairwise comparisons. As can be seen, no significant differences emerged between the two measures of happiness attainment (global and composite) used in the present study. By contrast, the mean global rating of unhappiness (*M* = 3.37, *SD* = 2.02) was significantly lower than the composite index of overall unhappiness (*M* = 4.09, *SD* = 2.23), *t*(170) = -4.31, *p* < .001. Furthermore, bivariate analyses conducted separately on all measures of overall happiness and unhappiness reported in Table 3 revealed no gender differences.

**Table 3:** Comparisons between Different Measures of Overall Happiness and Overall Unhappiness

	Global rating *M (SD*)	Composite index *M (SD)*	*t*
Overall happiness	6.98 (1.59)	7.14 (1.38)	-1.53
Overall unhappiness	3.37 (2.02)	4.09 (2.23)	-4.31*

* *p* < .001

### Further Analyses

In order to better understand the relationships between the various measures of overall happiness and overall unhappiness employed in the present study, correlational analyses were conducted. Results showed that global ratings and composite indexes were positively correlated for both happiness (*r* = .57, *p* < .001) and unhappiness (*r* = .48, *p* < .001). Moreover, whereas global ratings of happiness and unhappiness were negatively correlated (*r* = —.76, *p* < .001), there was not a significant correlation between the composite indexes of both investigated concepts (*r* = —.10, *p* = .89).

Finally, I computed, for each participant, two further indexes: The first one was obtained by summing the global ratings of happiness and unhappiness; on the other hand, the second index was calculated by summing the composite indexes of both concepts. Noteworthy, the decision to combine measures of overall happiness and overall unhappiness into sum scores was taken in order to increase the interpretability and meaningfulness of the new indexes. Indeed, both of them could range from 0 to 20, with values equal to 10 indicating perfect complementarity between happiness and unhappiness judgments. The analysis of frequency distributions of both indexes showed that 80.6% of participants obtained a value comprised between 9 and 11 on the first index, while only 36.5% of participants obtained a score comprised between these values on the second index. Overall, these results indicate that global ratings of happiness and unhappiness were roughly complementary, thus suggesting that participants did not contradict themselves when evaluating their overall subjective well-being. However, this was not the case when considering the composite indexes of overall happiness and unhappiness. Remarkably, the discrepancy between the frequency distribution of the first and second index may be due to the fact that the semantic components self-reported by participants were probably a limited subset of all aspects defining their ideas of happiness and unhappiness.

## Discussion

The present study aimed at investigating the subjective representation of happiness and unhappiness in a sample of Italian psychology undergraduates using a within-subjects research design. In line with previous investigations conducted on the same country (e.g., [25–27]), data were collected by means of a questionnaire including both open-ended and closed questions. This study thus followed a quali-quantitative approach as the free answers provided by participants were first coded on the basis of their semantic content and then subjected to statistical analyses.

With regard to the qualitative analyses conducted for the present work, the categorization of participants’ free answers yielded to the identification of 26 happiness components and 25 unhappiness components. Twenty-one of the semantic components defining happiness and unhappiness, respectively, referred to the same themes (e.g., health, love, money, friendship). However, the two investigated concepts also encompassed some distinctive components. Specifically, the characteristic components of happiness were *helping others*, *absence of unpleasant events*, *faith*, *pets*, and *luck*. On the other hand, the distinctive components of unhappiness were *death*, *loneliness* (cf. note 2), *interpersonal loss*, and *insecurity*.

The statistical analysis of frequency and order of citation of both happiness and unhappiness components permitted me to identify the core attributes defining the two concepts under investigation. According to the salience index–which combines information about these two statistical parameters–the most relevant components of the happiness concept were, in order, *family*, *friendship*, *love*, *health*, and *self-actualization*. Consistent with past studies conducted on Italian adult samples [25–27], participants mostly referred to life domains related to the satisfaction of basic biological and psychological needs. However, findings obtained in the present sample partially distinguish from previous ones as participants assigned greater importance to their friend relationships. In accordance with the arguments presented above, also these results may depend on the young age and the student status of subjects who participated in the present investigation. Indeed, both theory and empirical research suggest that building and cultivating relationships with friends significantly contributes to the young adults’ happiness and subjective well-being [39]. This has been found to be especially true for university students [40–41], also including psychology undergraduates [41].

With regard to the semantic features defining the unhappiness concept, its most salient components were, in order, *death*, *loneliness*, *health problems*, *bad social relationships*, and *failure*. As stated in the introduction, the only available data about folk psychology of unhappiness derive from a study by Uchida and Kitayama [31], who explored this topic in a sample of American and Japanese undergraduates. Some similarities emerge when comparing results from this cross-cultural study with the ones obtained in the present investigation. Specifically, in both studies participants associated the unhappiness concept with the occurrence of negative emotional experiences, personal failure, and bad social relationships. However, among the most salient unhappiness components reported by Italians there were some semantic categories which were not found in the Uchida and Kitayama’s study, namely *death*, *loneliness*, and *health problems*. Importantly, caution should be taken when comparing Uchida and Kitayama’s findings with the present ones. In fact, the two investigations employed different procedures of data collection and data analysis, which in turn influenced how researchers represented the semantic space of happiness and unhappiness. In particular, while Uchida and Kitayama identified a small set of categories defining the general meaning of both investigated concepts, the present study found a longer and more analytical list of semantic components.

Further indications about the semantics of happiness and unhappiness emerge when comparing the salience scores of happiness and unhappiness components referring to the same themes (e.g., *health* vs *health problems*). Significant differences were found in relation to seven themes. More in detail, the perceived salience of family, friendship, love, hobbies and interests, and partner was higher within happiness than unhappiness conceptions. On the other hand, participants considered everyday emotions and environmental mastery as themes more relevant for their unhappiness than for their happiness. Overall, these results suggest that interpersonal relationships with the social micro-environment were conceptualized by respondents as more important sources for their happiness than unhappiness. This was further corroborated by the finding that environmental mastery–which mainly referred to the relationship between participants and their social macro-environment–was a more salient theme within the unhappiness concept. Finally, differences in the salience scores associated with everyday emotions could be interpreted taking as reference the empirical literature on emotion lexicon. Indeed, a great deal of studies of both Anglo-Saxon and neo-Latin languages (including the Italian) have shown that that there are disproportionately more terms indicating negative emotions than positive ones ([42–45]; for a discussion of this topic, see [3]). I argue that this lexical disproportion may explain the greater salience emotions have within the unhappiness concept.

Correspondence analyses provided further insights about the semantic structure of participants’ conceptions of happiness and unhappiness. When examining the graphical output obtained for the happiness concept, two groups of components referring to the participants’ relational life are identifiable: The first one concerns the affective relationships with the partner and family members; on the other hand, the second one refers to friend relationships. Importantly, while both these life domains are core ingredients of participants’ representations of happiness, they seem to be conceptualized as two separate aspects of human existence, the second one being connected to personal and social success. Also in this case, results may be ascribed to the young age of participants, and specifically to their desire to manage friend relationships autonomously. However, further studies using qualitative methods are needed to clarify how young adults attending university differentially link the multiple aspects of relational life with the their ideas of happiness and unhappiness.

Another interesting result emerging from correspondence analysis regards the spatial proximity between the components *death* and *loneliness* within the semantic map of unhappiness. Noteworthy, the mean presence in life scores computed for both components were quite low (i.e., below the midpoint of the scale). Since it can be assumed that actual experiences of death and loneliness did not occur frequently in the life of participants, other factors should explain the close relationship between these two components. In my opinion, the most plausible one is that both death and loneliness are core themes of culturally shared representations of unhappiness. Indeed, Western popular culture–as reflected in movies, novels, songs, and the like–typically emphasizes a strong connection between the death of significant others, feelings of loneliness, and unhappiness (on this topic, see [46]). Italian psychology students might not be immune to these cultural influences.

Beside exploring the semantics of happiness and unhappiness, the questionnaire employed in the present study allowed me to assess to what extent participants felt happy and unhappy when thinking about their life. Different measures of overall happiness and overall unhappiness were used: Global and composite. The comparison between them showed that participants assessed their life as less unhappy when providing a global evaluation about it as opposed to when they rated a list of specific self-reported unhappiness components. What do these results tell us about the subjective well-being of participants involved in the present study?

In answering this question, we should keep in mind that global and specific measures of happiness and unhappiness are assumed to capture different information about people’s lives. As suggested by several authors [47–50], global reports typically reflect moods and dispositional tendencies; by contrast, specific measures of well-being are more tied to people’s actual experiences in multiple life domains. Based on these premises, the results of the present study showed that while global reports of happiness accurately reflected participants’ everyday experiences, global evaluations of unhappiness did not. Rather, the latter offered a more positive image of these experiences. A possible interpretation of this judgmental asymmetry points to the effects of a social desirability bias. Indeed, the subjective tendency to formulate and express a positive evaluation of oneself might have a stronger influence on global unhappiness judgments than on global happiness judgments. This issue warrants further investigation.

## Limitations and Future Research Directions

The present study suffers from two main limitations. The first one concerns the characteristics of the investigated sample. Indeed, only Italian psychology undergraduates, mostly females, took part in the study. The generalizability of findings to other populations is thus strongly limited. On the other hand, a second limitation concerns the empirical assessment of participants’ levels of happiness and unhappiness. As it has been previously said, different measures of overall happiness and overall unhappiness were used in the present study. However, since all of them were obtained from an *ad hoc* questionnaire, I did not have the opportunity to examine their correlation with well-validated measures of happiness and unhappiness.

Based on all these considerations, I suggest that future research on conceptions of happiness and unhappiness would follow three main directions. First, researchers should made an attempt at replicating the present findings by surveying psychology undergraduates from other countries and socio-cultural contexts. This would make it possible to detect cross-cultural similarities and differences in the conceptions of happiness and unhappiness, thus expanding the current literature on this topic. Second, it would be important for future researchers to examine large nonstudent samples from the general population, adequately representing genders, age groups, educational levels, and income ranges. This would allow researchers to assess sociodemographic differences in the subjective representation of happiness and unhappiness, which were not captured by the present investigation. Third, future researchers might integrate the use of *ad hoc* questionnaires, providing both qualitative and quantitative data, with standardized scales providing only quantitative information. Indeed, the current literature encompasses several psychometrically valid instruments which quantitatively assess various forms of psychological well-being: For example, hedonic well-being [51], eudaimonic well-being [52], and social well-being [53]. I argue that investigating the relationship between measures deriving from these instruments and quantitative assessments taking into account participants’ lay conceptions–such as the ones employed in the present study–would increase the validity of studies on folk psychology of happiness and unhappiness (on this topic, see [28]).

## Conclusion

In accordance with the study by Uchida and Kitayama [31], the results of the present investigation suggest that participants did not conceptualize happiness and unhappiness as perfect antonyms. Indeed, both investigated concepts encompassed a similar set of semantic components; however, the perceived salience of some of them significantly varied between happiness and unhappiness. Furthermore, some semantically distinct components were found within the participants’ representations of the two concepts.

When looking at these results, we should keep in mind that psycholinguistic research on Anglo-Saxon and neo-Latin languages–also including the Italian [14–15, 18]–demonstrated that happiness constitutes a more prototypical emotional category than unhappiness, being used more frequently in everyday language. I argue that the higher prototypicality of the happiness concept, along with the fact that subjects who took part in the present study considered themselves as moderately happy (and only slightly unhappy), may explain the differences which were found in the subjective representations of the two concepts under investigation. Naturally, this interpretation is proposed taking into account both the linguistic context and the specific characteristics of the sample examined in the present study. Further research investigating other languages and cultural contexts, as well as samples of participants reporting lower levels of subjective well-being, is needed in order to better understand the folk psychology of happiness and unhappiness.
